# Inhaled Cadmium Oxide Nanoparticles: Their *in Vivo* Fate and Effect on Target Organs

**DOI:** 10.3390/ijms17060874

**Published:** 2016-06-03

**Authors:** Jana Dumkova, Lucie Vrlikova, Zbynek Vecera, Barbora Putnova, Bohumil Docekal, Pavel Mikuska, Petr Fictum, Ales Hampl, Marcela Buchtova

**Affiliations:** 1 Department of Histology and Embryology, Faculty of Medicine, Masaryk University, Brno 625 00, Czech Republic; jdumkova@med.muni.cz (J.D.); gahampl@gmail.com (A.H.); 2 Institute of Animal Physiology and Genetics, v.v.i., Czech Academy of Sciences, Brno 602 00, Czech Republic; vrlikova@iach.cz (L.V.); bara.putnova@gmail.com (B.P.); 3 Institute of Analytical Chemistry, v.v.i., Czech Academy of Sciences, Veveří 97, Brno 602 00, Czech Republic; vecera@iach.cz (Z.V.); docekal@iach.cz (B.D.); mikuska@iach.cz (P.M.); 4 Department of Pathological Morphology and Parasitology, Faculty of Veterinary Medicine, University of Veterinary and Pharmaceutical Sciences, Brno 612 42, Czech Republic; Petr.Fictum@seznam.cz; 5 Department of Animal Physiology and Immunology, Faculty of Science, Masaryk University, Brno 625 00, Czech Republic

**Keywords:** nanoparticles, cadmium oxide, electron microscopy, toxicity, inhalation, lung, liver, kidney, spleen

## Abstract

The increasing amount of heavy metals used in manufacturing equivalently increases hazards of environmental pollution by industrial products such as cadmium oxide (CdO) nanoparticles. Here, we aimed to unravel the CdO nanoparticle destiny upon their entry into lungs by inhalations, with the main focus on the ultrastructural changes that the nanoparticles may cause to tissues of the primary and secondary target organs. We indeed found the CdO nanoparticles to be transported from the lungs into secondary target organs by blood. In lungs, inhaled CdO nanoparticles caused significant alterations in parenchyma tissue including hyperemia, enlarged pulmonary septa, congested capillaries, alveolar emphysema and small areas of atelectasis. Nanoparticles were observed in the cytoplasm of cells lining bronchioles, in the alveolar spaces as well as inside the membranous pneumocytes and in phagosomes of lung macrophages. Nanoparticles even penetrated through the membrane into some organelles including mitochondria and they also accumulated in the cytoplasmic vesicles. In livers, inhalation caused periportal inflammation and local hepatic necrosis. Only minor changes such as diffusely thickened filtration membrane with intramembranous electron dense deposits were observed in kidney. Taken together, inhaled CdO nanoparticles not only accumulated in lungs but they were also transported to other organs causing serious damage at tissue as well as cellular level.

## 1. Introduction

Cadmium as a chemical element (transitional metal) is chemically similar in many aspects to the two other stable metals in its group, zinc and mercury. Cadmium makes up only about 0.1 ppm of Earth’s crust, compared with the more abundant 65 ppm zinc [1]. Cadmium has no known biological function in higher organisms. Some metals, such as copper (Cu) or zinc (Zn), are essential micronutrients, although they are also toxic in higher concentrations. On the other hand, metals such as cadmium, lead and mercury can damage numerous biochemical pathways, even at low concentration [2].

Air, soil and water metal pollution results dominantly from the emission of fumes and smoke from industrial plants and automobile exhaust fumes. Following wet or dry deposition, these metals remain in the soil and they are transferred from contaminated soils into the plant tissue and via food chain endanger the human health. The positive relation of higher metal concentration of metals in the plants (both widely spread species and vegetables) with local environmental pollution has been unraveled by many previous studies [3,4,5]. Bio-concentration factor values for Cd were found to be higher than those for, e.g., Pb and Cr, which indicate that cadmium is more readily absorbed by vegetables than lead and chromium. Therefore, more attention should be paid to the possible toxicity of cadmium [3,4,5].

Another exposure risk to the biota is metal contamination from the environment and inhalation of these metals. This exposure has increased dramatically during the first industrial revolution due to anthropogenic sources such as combustion engines or power plants. Today, new technology processes produce the nanoscale metal objects that are being incorporated within a wide range of applications: biomedical, cosmetic, car-parts, food, energy production and electronics [6]. Due to this fact and the reality that the one-half of the World’s population now lives in urbanized areas, metals continue to present a serious issue for public health [2].

Cadmium is a toxic metal found in the environment naturally and as a pollutant emanating from industrial and agricultural sources. It is an extremely toxic element of ongoing concern because its environmental levels have risen steadily due to continued worldwide anthropogenic mobilization and because of its diverse toxic effects: extremely protracted biological half-life (approximately 20–30 years in humans), low rate of excretion from the body, and storage predominantly in soft tissues (primarily liver and kidneys) [7]. Another important source of cadmium exposure is tobacco smoking. It was estimated that a person smoking 20 cigarettes a day will absorb about 1 µg of cadmium. Food is the main source of cadmium intake in the non-smoking population [8,9].

Nanoparticles (NPs) are generally characterized by diameter smaller than 100 nm. Their chemical composition, type, particle size, coating, charge and concentration may all influence their ultimate effect on humans and animals. Inhalation is the most frequent type of entry of nanoparticles to the body and it causes more severe acute damage or chronic problems both in lungs [10,11,12] and other organs in body [13,14,15]. It has been shown that inhaled nanoparticles (NPs) are able to pass through the alveolar epithelium of the lungs and endothelial cell layer into the blood stream and thus influence other organs [16]. Nano-sized metals, compared to conventionally sized particles, are of particular risk since they more easily enter lung tissues after inhalation.

Our study seeks further expansion of the understanding of nanoparticle fate upon their entry into lungs, with the main focus on the changes that the NPs may cause to target tissue structure. Cadmium has numerous undesirable effects in both humans and animals, such as toxic effects including nephrotoxicity, carcinogenicity, teratogenicity and endocrine and reproductive toxicities [10,11,12]. At the cellular level, cadmium affects cell proliferation, differentiation, apoptosis and it can induce genomic instability [7]. Since nanoparticles containing cadmium (Cd) present a serious issue for human health, cadmium oxide (CdO) nanoparticles were chosen for the evaluation.

## 2. Material and Methods

### 2.1. Animals

Adult female mice (ICR strain) were obtained from the Animal facility of the Masaryk University (Brno, Czech Republic). Animals were allowed to acclimate to laboratory conditions for at least 1 week before the inhalation experiments. Commercial diet and water were provided *ad libitum*. The experiments were performed in accordance with the ethical approval of the Institute of Animal Physiology and Genetics (No. 081/2010; 29 March 2010).

### 2.2. Preparation of CdO Nanoparticles

CdO nanoparticles (CdONPs) were generated continuously *in situ* in a hot wall tube flow reactor using an evaporation–oxidation–condensation technique in which a ceramic crucible containing a small amount of granular cadmium was placed inside the ceramic work tube of a vertically orientated furnace (Carbolite TZF 15/50/610). The melted cadmium was evaporated at the center of the furnace at a temperature of 340 °C. Formed metal vapor was carried out of the furnace with an inert nitrogen gas stream and diluted with a stream of air when oxygen oxidized the cadmium to cadmium oxide. Both flow rates were set at 3 L/min with mass flow controllers (MFC). The formed cadmium oxide nanoparticles were diluted in the second step with a stream of air (20 L/min) and used for whole body inhalation experiments in exposure chambers.

### 2.3. Characterization of Generated CdO Nanoparticles

The distribution of nanoparticles (NPs) with respect to number concentration of particles was measured directly using a Scanning Mobility Particle Sizer (model 3936L72, TSI Inc., Shoreview, USA TSI; Differential Mobility Analyzer model 3081 and Condensation Particle Counter model 3772) continuously in the size range of 7.64–229.6 nm (Figure S1). Size distribution of smaller nanoparticles down to 2.02 nm was measured in a few experiments using a nanoDMA (model 3085, combined with CPC, model 3775, both from TSI). Combination of size distributions measured by both size instruments is shown in Figure S2.

The long-term stability of NPs generation was high. The relative standard deviations in median particle diameter (*i.e.*, 12.3 nm) and total particle concentration (*i.e.*, 2.95 × 10^6^ particles/cm^3^) were 5.5% and 4.8%, respectively. These values indicate that both the generation of NPs and the measurement of size distribution using the Scanning Mobility Particle Sizer were very reproducible.

Mass concentration of generated CdO nanoparticles was calculated by dividing of mass of CdO nanoparticles collected on filter by volume of air sample that was passed through the filter. Generated CdO nanoparticles were sampled on nitrocellulose filters (pore size 0.45 µm, diameter 25 mm, Millipore, Bedford, MA, USA), filters were decomposed in HNO_3_ using UniClever microwave mineraliser (Plazmatronika, Wroclaw, Poland) and content of Cd in decomposed sample was analyzed by means of AAS (AAnalyst 600, PerkinElmer Inc., Shelton, CT, USA).

Size and shape of generated CdO nanoparticles were measured by electron microscopy. CdO NPs immediately after their generation at the output from the furnace were collected by electrostatic precipitation using a Nanometer aerosol sampler (model 3089, TSI) on TEM grids (copper S160-4, 3 mm in diameter, 400 mesh grids, Agar Scientific, Electron Technology, Stansted, Essex, UK). The samples were analyzed by means of the Magellan 400 L XHR microscope (FEI Company, Hillsboro, OR, USA) operating in the STEM mode. The micrograph (Figure S3A–D) shows that the CdO NPs observed in the gas phase by SMPS analysis are formed by agglomerates of primarily 2–7 nm in diameter sized particles.

### 2.4. Exposure to CdO Nanoparticles

The inhalation chamber was made of glass and stainless steel and contained two stainless steel inhalation cages (control and cadmium treated). Air-conditioning system maintained constant parameters of air that was passed through the inhalation cages (flow rate, temperature, relative humidity and pressure). Actual levels of the air parameters were on-line measured and recorded in 1-min periods. Flow rate ranged between 19.9 and 20.1 L/min, temperature ranged between 20.2 and 21.5 °C, relative humidity ranged between 58% and 68% and pressure was in the range of 995–1020 hPa. The illumination period was set to 12 h light and 12 h dark. Behavior and health condition of mice during the experiments were continuously monitored by camera system.

Adult female mice with average weight about 24 g were continuously exposed to the CdO nanoparticles for 6 weeks (for 24 h/day, 7 days/week). Control animals were exposed to the same air as the experimental group just without nanoparticles supplement. Four biological replicates (individuals) were used for each treatment.

Mice were separated into two groups for each exposure time: a fresh-air control group (co) and group exposed to CdO nanoparticles. This group (Cd) was exposed to average number concentration of 2.95 × 10^6^ particles/cm^3^ (mode 9.82 nm, geometric mean diameter 15.3 nm, median 12.3 nm, geometric standard deviation 1.61, average mass concentration of CdO nanoparticles was 31.7 µg CdO/m^3^). Estimated deposited dose was 52 µg of CdO per gram of mouse body weight over the 6 weeks inhalation period [17,18,19].

At the end of exposure, mice were sacrificed by cervical dislocation. Organs were collected for biochemical, histological and electron microscopic analyses. The following organs were collected and analyzed: lung, liver, kidney and spleen.

### 2.5. Histological Analysis

Samples of organs were fixed in 10% buffered neutral formaldehyde for histological analysis. Following overnight fixation in fridge, organ samples were dehydrated in increasing series of ethanol, treated by xylene and embedded in paraffin. Serial histological sections of 5 µm thickness were prepared and selected slides were stained in Hematoxylin-Eosin and Green Trichrome using standard histological techniques. Sections were examined by light microscopy in a blinded fashion.

### 2.6. Transmission Electron Microscopy

Organ samples were fixed in 3% glutaraldehyde −0.1 M cacodylate buffer for 24 h. After three washing steps in 0.1 M cacodylate buffer, samples were post-fixed in 1% OsO_4_ solution in the same buffer for 1 h. The specimens were then dehydrated in ethanol, treated with acetone and embedded in epoxy resin Durcupan. Semithin sections were stained with Toluidine Blue. Thin sections were cut at 60 nm on an ultramicrotome Leica EM UC6 (Leica Mikrosysteme GmbH, Vienna, Austria) and placed on 50 mesh formvar-coated nickel grids.

Selected ultrathin sections were contrasted with 2.5% uranyl acetate for 10 min and alkaline Reynolds lead citrate solution for 6 min, the other sections were used without any further contrasting, the sections were observed using Morgagni™ 268 TEM (FEI Company, Eindhoven, The Netherlands). The pictures were taken with Veleta *CCD* Camera (Olympus, Münster, Germany).

### 2.7. Analysis of Cd Content in Mouse Organs

The aliquots of individual organs of maximum weight of 1 g were decomposed in 3 mL of concentrated sub-boil grade (quartz distillation system model MSBQ 2, Maasen, Eningen, Germany) nitric acid by microwave (MW) assisted digestion. The samples were treated in pre-cleaned PTFE vessels of closed pressurized autoclave system (UniClever, Plazmatronika, Wroclaw, Poland). The decomposition program consisted of three steps, each in duration of 5 min, applying 70, 90 and 100 percent of microwave power (100 W) controlling working pressure within limits of 35/32, 40/35 and 45/42 bar, respectively. After cooling down (10 min), digests were quantitatively transferred to pre-cleaned PP vials, diluted and adjusted with ultrapure water (Ultra Clear system, SG Water Systems, Barsbüttel, Germany) to the final mass of 10 g. Simultaneously, blank samples (typically *n* = 12 per sampling series) were processed the same way as samples.

The content of cadmium in digests was determined by electrothermal atomic absorption spectrometry (ETAAS) employing AAnalyst 600 (PerkinElmer Inc., Shelton, CT, USA) instrumentation under recommended procedure. The mixture of ammonium phosphate and magnesium nitrate was used as a combined chemical modifier. Method of standard addition calibration was applied for quantitation. The content of cadmium was determined in lungs, liver, kidney and spleen of mice individuals exposed to 6 weeks of inhalation.

### 2.8. Analysis of Mouse Blood for Cd Content

Blood was collected by cardiac puncture into 1 mL plastic Eppendorf tubes with small amount of heparin. Blood samples were divided into three phases. After centrifugation blood elements were separated, by force of methanol (300 µL) were proteins separated from remaining supernatant. Blood samples were stored in a refrigerator at 5 °C before subsequent analyses.

Individual phases of blood were decomposed by means of ozone and nitrous oxides gas mixture employing system Dry Mineralizer Apion (Tessek, Prague, Czech Republic) under atmospheric pressure. Blood samples were quantitatively transferred to special pre-cleaned glass vials, 1 mL of 1 M nitric acid in high purity sub-boil was added to every sample for better mineralization. Vials were embedded in thermo-block heating system, in which the samples are at first dehydrated at 110 °C (rump time 45 min) during 60 min, then the samples are mineralized at 380 °C (rump time 4 h) for a period 8 h with synchronic gas mixture introduction. After cooling of vials, sample digests were diluted in 1 mL 1 M nitric acid of sub-boil purity, quantitatively transferred into plastic scintillation vials, adjusted with ultrapure water (Ultra Clear system, SG Water Systems, Barsbüttel, Germany) to the final mass of 3 g and stored for the next analysis. Simultaneously, blank samples were processed the same way as blood samples.

The content of cadmium in blood digests was determined by electrothermal atomic absorption spectrometry (ETAAS) employing AAnalyst 600 (PerkinElmer Inc., Shelton, CT, USA) instrumentation under recommended procedure. The mixture of ammonium phosphate and magnesium nitrate was used as a combined chemical modifier. Method of standard addition calibration was applied for quantitation.

### 2.9. Data Analysis

Results are presented as the mean ± standard deviation of the mean. Data were analyzed using the Statistica 8.0 software (StatSoft. Inc., Tulsa, OK, USA). One-way analysis of variance was performed for overall comparisons, while the Student–Newman–Keuls *post hoc* test was used to determine differences between groups. Values of *p* < 0.05 were considered to be statistically significant.

## 3. Results

### 3.1. The Effect of Inhaled Nano-Sized Particles of Cadmium Oxide on Mouse Lungs—As a Primary Target Organ

**Figure 1:**
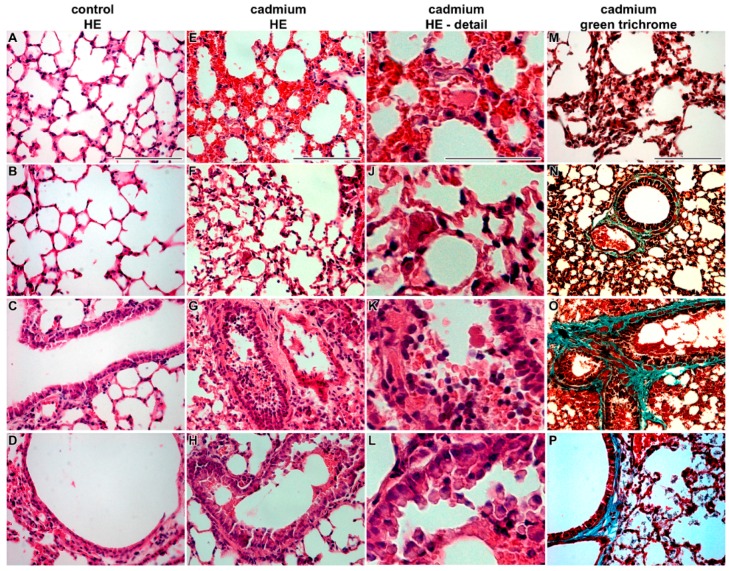
Effect of cadmium nanoparticles on lung tissue following six weeks exposure to CdO nanoparticles: (**A**–**D**) control tissues; and (**E**–**P**) cadmium nanoparticles exposed tissues. Hematoxylin-Eosin and Green Trichrome staining. Scale bar: **A**–**H** = 100 μm, **I**–**L** = 50 μm, **M**–**P** = 100 μm.

Histopathological analysis revealed that CdO nanoparticles caused severe alterations in lung morphology following sub-chronic exposure. Histopathological changes in lungs were visible both in the respiratory passages as well as in the alveoli. After six weeks of cadmium exposure, the lumen of bronchioli was filled by mucous secretion, desquamated epithelial cells, and inflammatory cells—predominantly neutrophils and sporadic red blood cells (Figure 1). Affected lungs exhibited light hyperemia, congested capillaries, alveolar emphysema and small areas of atelectasis. Increased numbers of neutrophils and macrophages were found in alveoli.

**Figure 2:**
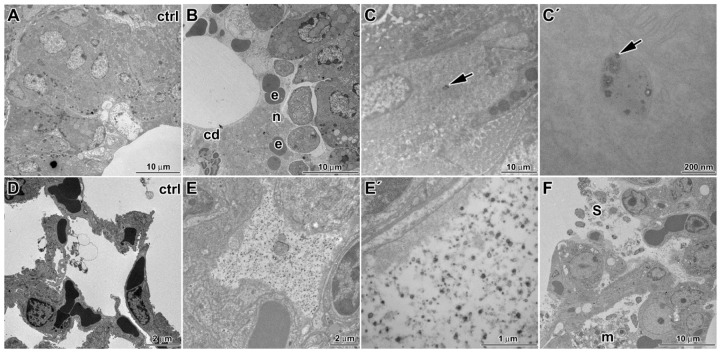

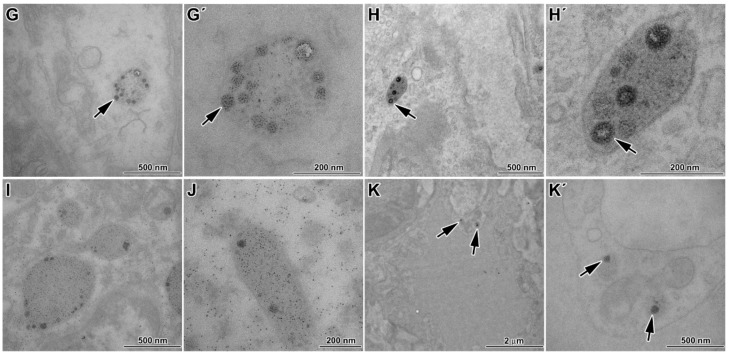
Ultracellular changes in lung tissue following six weeks exposure to CdO nanoparticles: (**A**) bronchiole in control sample; (**B**) bronchiole after treatment with signs of inflammation, neutrophils (n), erythrocytes (e) and cell debris (cd) present in lumen of bronchiole; (**C**,**C′**) bronchiolar epithelium with vesicle full of different sized cadmium nanoparticles; (**D**) alveoli in control sample; (**E**,**E′**) alveoli after treatment are completely filled with nanoparticles; (**F**) in alveoli, higher amount of surfactant (s) and impaired alveolar macrophage (m) is obvious; (**G**,**G′**,**H**,**H′**) cadmium nanoparticles in vesicles of alveolar epithelial cells type I; (**I**,**J**) cadmium nanoparticles in phagosomes of pulmonary macrophage; and (**K**,**K′**) cadmium nanoparticles in vesicles of capillary endothelial cell. Arrows show nanoparticles.

Using transmission electron microscopy, we found additional changes such as edematous enlargement of membranous pneumocytes and endothelial cells in some regions (Figure 2). There was also a presence of apoptotic and necrotic cells (mostly macrophages and granular pneumocytes). The abruption and fragmentation of the alveolar epithelial type I cells and endothelial cells of capillaries with denuded basement membrane was detected. Furthermore, enlarged pulmonary septa were observed.

Nanoparticles were enveloped in surfactant of the alveoli. Notably, increased amount of tubular myelin (intermediate step in the formation of surface film) was indicative of enhanced production of surfactant by alveolar epithelial type 2 cells (PII). Frequent plasma cells were concentrated in the interstitium.

In bronchioli, excessive production of secretions from club cells were observed and they contained increased number of nucleoli in nuclei indicating higher metabolic activity of these cells. Furthermore, higher presence of neutrophils, macrophages and frequent erythrocytes were found in luminal parts of bronchioli with signs of disintegration or necrosis. Bronchiolar epithelium of some bronchioli also exhibited signs of necrosis (predominantly nuclei and apical portions of cells).

In summary, the inhalation of CdO nanoparticles caused morphological changes of lung tissue that are typical for inflammation.

### 3.2. The Effect of Inhaled Nano-Sized Particles of Cadmium Oxide on Mouse Liver—As a Secondary Target Organ

**Figure 3:**
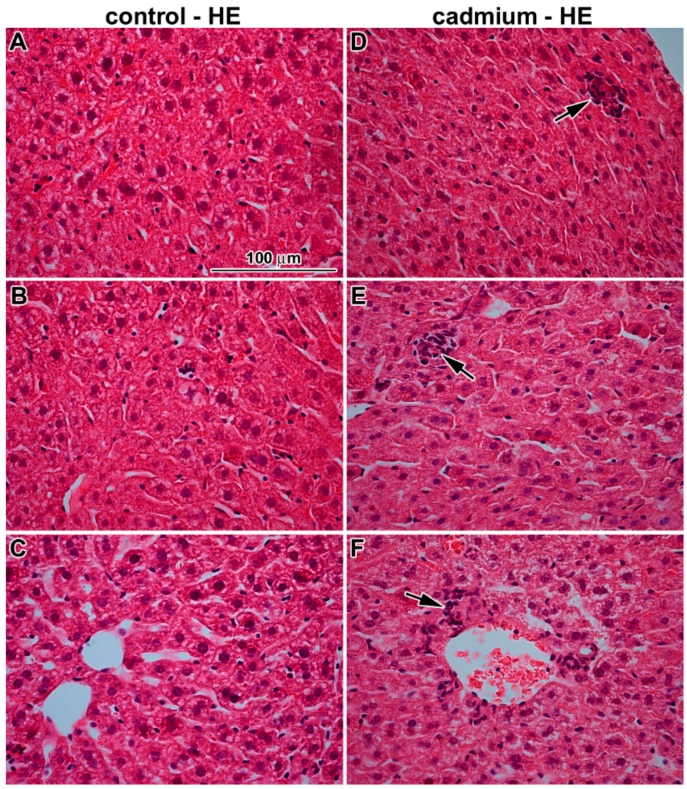
Effect of cadmium nanoparticles on liver following six weeks exposure to CdO nanoparticles: (**A**–**C**) control tissues; and (**D**–**F**) cadmium nanoparticles exposed tissues. Hematoxylin-Eosin. Scale bar = 100 μm. Arrows show small areas of necrosis.

Histopathological analysis revealed that inhaled CdO nanoparticles caused severe alterations in liver morphology following sub-chronic exposure (Figure 3). Typically, there were areas of periportal inflammation, with their number as well as areas being increased with the length of exposure to CdO nanoparticles. Occasional neutrophilic granulocyte infiltrations around vessels and small areas of hepatic necrosis were observed in some samples.

Hepatocytes around central vein were without obvious pathological changes resembling the controls. These hepatocytes had the same size as hepatocytes in the control samples, their nuclei (1 or 2) were round, small with smooth contours, contained few nucleoli, and euchromatin was prevalent by contrast to hepatocytes next to the periphery of hepatic lobule. Hepatocytes of zone 1 of liver acinus (closest to the arterioles) are the best oxygenated and they were the first to absorb blood-borne nanoparticles. These cells exhibited typical signs of necrosis.

**Figure 4:**
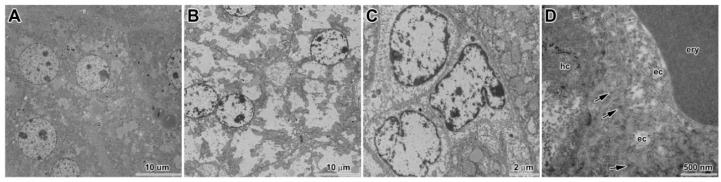

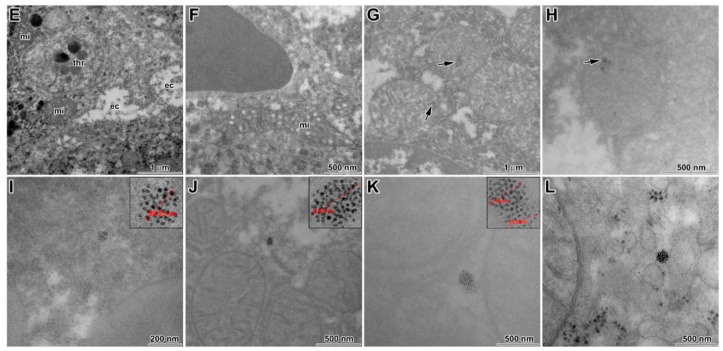
Ultracellular changes in liver following of six weeks exposure to CdO nanoparticles: (**A**) hepatocytes after treatment without pathological changes; (**B**) hepatocytes after treatment with altered ultrastructure showing severe signs of degeneration; (**C**) epithelial cells of bile duct with signs of hydropic degeneration; (**D**) microvilli (arrows) of hepatocyte in Disse’s space, cytoplasmic parts of edematous endothelial cells, erythrocyte in sinusoid capillary; (**E**) mitochondria in Kupffer cell with unusual arrangement of mitochondrial cristae, cytoplasmic parts of edematous endothelial cells, thrombocyte in lumen; (**F**) mitochondria in Kupffer cell with unusual arrangement of mitochondrial cristae; (**G**,**H**) mitochondria in hepatocytes with observed cadmium nanoparticles (arrows) enveloped in denser mitochondrial matrix; and (**I**–**L**) cadmium nanoparticles freely in cytoplasm of hepatocytes: (**I**–**K**) non-contrasted sections; and (**L**) contrasted section. ec, endothelial cells; ery, erythrocyte; hc, hepatocyte; mi, mitochondria; thr, thrombocyte.

Periportal hepatocytes were larger with obvious damage, they exhibited clear round nuclei, their cytoplasm looked somewhat empty, cell organelles were degraded or showed severe signs of pathological processes including hydropic dystrophy (ruptures of its membrane, presence of vacuoles in their bodies, and edematous enlargement) (Figure 4).

The epithelial cells of bile duct were also affected. Nuclei and cytoplasm of these cells showed signs of necrosis. The hepatic sinusoids were destroyed similarly to bile ducts. Contrary to this observation, the number of Kupffer cells and Ito cells remained unaffected. In Kupffer cells, we observed unusual mitochondria with tubules and dilated cisternae of smooth endoplasmic reticulum possibly indicating altered immune (or steroidogenic) function. Blood in hepatic sinusoids contained higher amount of reticulocytes (typical sign of enhanced erythropoiesis).

### 3.3. The Effect of Inhaled Nano-Sized Particles of Cadmium Oxide on Mouse Kidney and Spleen—As a Secondary Target Organs

**Figure 5:**
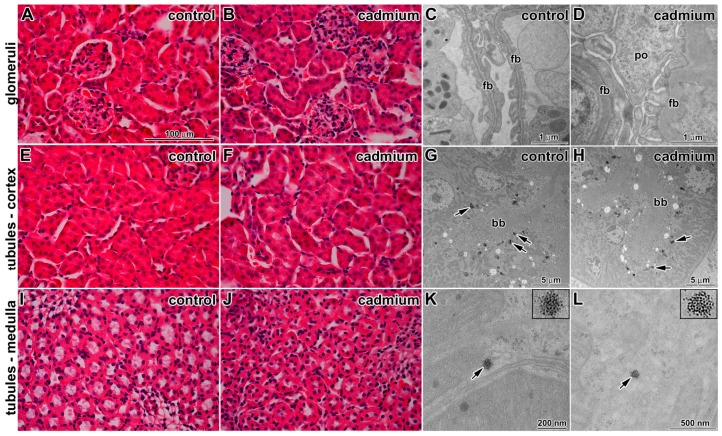
Effect of cadmium nanoparticles on kidney following six weeks exposure to CdO nanoparticles: (**A**) glomerulus in control sample (Hematoxylin-Eosin); (**B**) glomerulus in cadmium treated sample (Hematoxylin-Eosin); (**C**) filtration barrier is thin in control sample; (**D**) alteration of filtration barrier (fb) morphology after treatment, body of podocyte (po); (**E**) unaffected tubules in control tissue (Hematoxylin-Eosin); (**F**) tubules in the kidney cortex of cadmium treated animal (Hematoxylin-Eosin); (**G**) proximal tubule in control sample, (bb) signs of brush border, arrows show secondary lysosomes with dense granular content; (**H**) proximal tubule after cadmium treatment, (bb) signs of brush border, arrows show secondary lysosomes with dense granular content without nanoparticles; (**I**) unaffected tubules in control tissue of medulla (Hematoxylin-Eosin); (**J**) tubules in the kidney medulla of cadmium treated animal (Hematoxylin-Eosin); and (**K**,**L**) cadmium nanoparticles (arrows) freely in epithelial cell cytoplasm of proximal tubules. Scale bar A,B,E,F,I,J = 100 μm.

Exposure to CdO nanoparticles caused no or only minor changes to kidney ultrastructure (Figure 5). Renal tubules (proximal convoluted (PT), distal convoluted (DT), and loop of Henle) as well as cortical and medullary collecting ducts had standard compositions. Some apoptotic cells were observed in tubules; however, their amount was similar to the control samples.

In renal corpuscles, cytoplasmic processes of intraglomerular mesangial cells were located among processes of podocytes. We noted differences in arrangement of filtration barrier. In CdO treated samples, lamina rara subendothelialis and lamina rara subepithelialis were filled by fine deposits electron dense material. Lamina densa was thicker in comparison to the control. The contrast among these layers (laminae) disappeared after cadmium exposure (Figure 5).

In consonance with the effects described above, we observed diffusely thickened filtration membrane with intramembranous electron dense deposits. The distance between cytoplasmic membrane of endothelial cells and cytoplasmic membrane of podocytes in the controls was about 140–150 nm. This distance was increased to more than 200 nm in animals exposed to the CdO nanoparticles. Higher amount of thrombocytes was found in glomerular capillaries in comparison to the control samples. This may correspond to increased thrombopoiesis in spleen.

**Figure 6:**
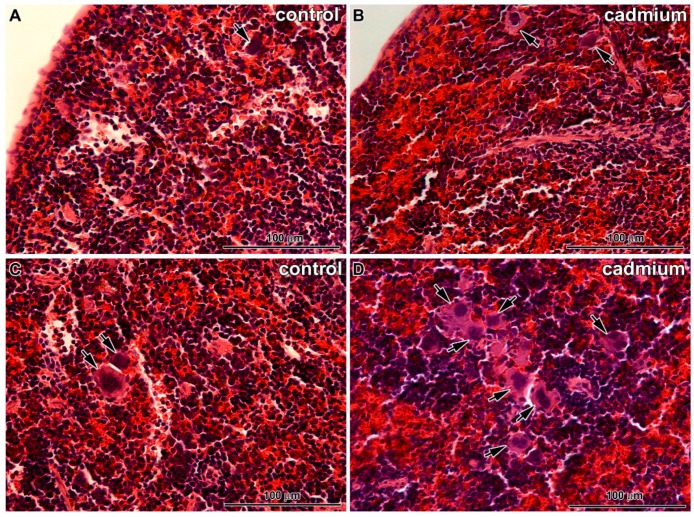

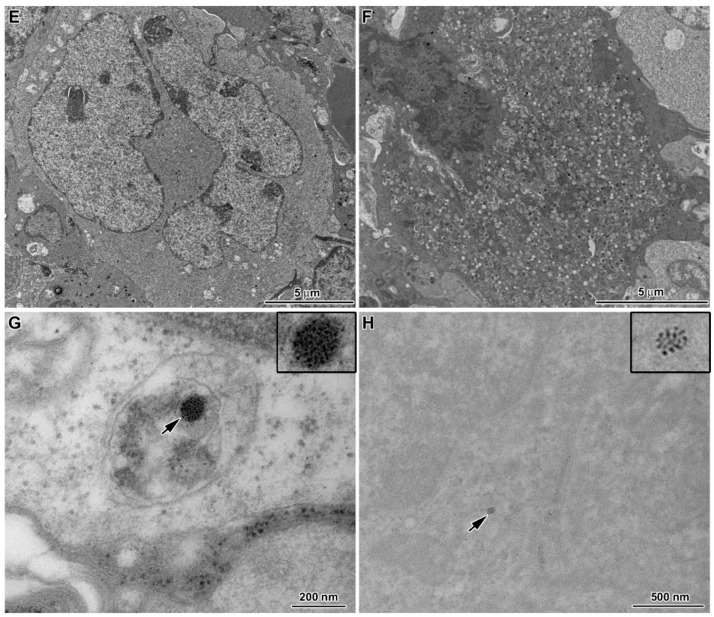
Effect of cadmium nanoparticles on spleen following six weeks exposure to CdO nanoparticles: (**A**) superficial area of control tissue; (**B**) superficial area of spleen after cadmium treatment; (**C**) deeper area of control tissue; (**D**) deeper area of spleen after cadmium treatment (**A**–**D**) Hematoxylin-Eosin, scale bar = 100 μm; (**E**) megakaryocyte in control sample; (**F**) apoptotic megakaryocyte after cadmium treatment; (**G**) cadmium nanoparticles in vesicle of lymphocyte (contrasted section); and (**H**) cadmium nanoparticle freely in cell cytoplasm (non-contrasted section). (**A**–**D**) Arrows show megakaryocytes. (**G**,**H**) Arrows show cadmium nanoparticles.

Only fine changes were observed in the spleens of animals exposed to CdO nanoparticles (Figure 6). There were visible typical lienal structures similar to the controls, an arrangement of red pulp and white pulp with vessel supply as well as areas of erythroblastic islands and regions of granulopoiesis were present. The only exception was small incensement of the number of megakaryoblasts and megakaryocytes in red pulp. We also observed some apoptotic events of these thrombopoetic cells in transmission electron microscope (Figure 6).

### 3.4. Cellular Uptake of Inhaled CdO Nanoparticles in Lungs

Cellular uptake of nanoparticles was analyzed in animals that were exposed to cadmium for six weeks. CdO nanoparticles accumulated in the alveolar spaces as well as inside the cells. Nanoparticles were observed in alveolar epithelial type I cells (membranous pneumocytes (PI)) but not in alveolar epithelial type II cells (granular pneumocytes (PII)). Nanoparticles were also located in phagosomes of lung macrophages (Figure 2). Here, nanoparticles formed electron dense clusters enveloped by cytoplasmic membrane, thus forming numerous distinct cytoplasmic vesicles.

We also found the presence of CdO nanoparticles in cytoplasm of cells lining bronchioles. Thus, nanoparticles can pass through the epithelial cells in all levels of respiratory passages, before reaching the pulmonary alveoli. In these cells, nanoparticles were situated inside large cytoplasmic vesicles in clusters. These vesicles exhibited appearance typical for caveolae.

There were no cadmium nanoparticles found in lungs of control mice, which had been exposed just to clean aerosol.

### 3.5. Cellular Uptake of Inhaled CdO Nanoparticles in Liver

Following six weeks of inhalation, CdO nanoparticles were found in cytoplasm of hepatocytes (Figure 4). Numbers of nanoparticles varied from solitary to moderate. Nanoparticles were usually found localized as single clusters of primary particles, doublets of clusters or agglomerates of several clusters close to each other. They were not surrounded by any membrane and were not in contact with any organelles. Nanoparticles were rarely found inside hepatic mitochondria. In these regions, nanoparticles were observed in groups and enveloped by electron dense mitochondrial matrix.

CdO nanoparticles were not observed in any other types of hepatic cells such as Kupffer cells, Ito cells, inside vessels or bile ducts. Nanoparticles did not enter nuclei of any type of examined liver cells.

### 3.6. Cellular Uptake of Inhaled CdO Nanoparticles in Kidney and Spleen

In kidneys, the nanoparticles were only rarely present as clusters located inside the cytoplasm of the epithelial cells in the proximal tubules of kidney cortex (Figure 5). No particles were found inside the nucleus of these cells. In other kidney regions, we did not observe any nanoparticles.

Similarly, the presence of nanoparticles in spleen was also very rare, being distributed in cytoplasm or in cytoplasmic vesicles. Moreover, they were also present in lymphocytes (Figure 6).

### 3.7. Content of Cadmium in Organs and Blood Following CdO Exposure

**Figure 7:**
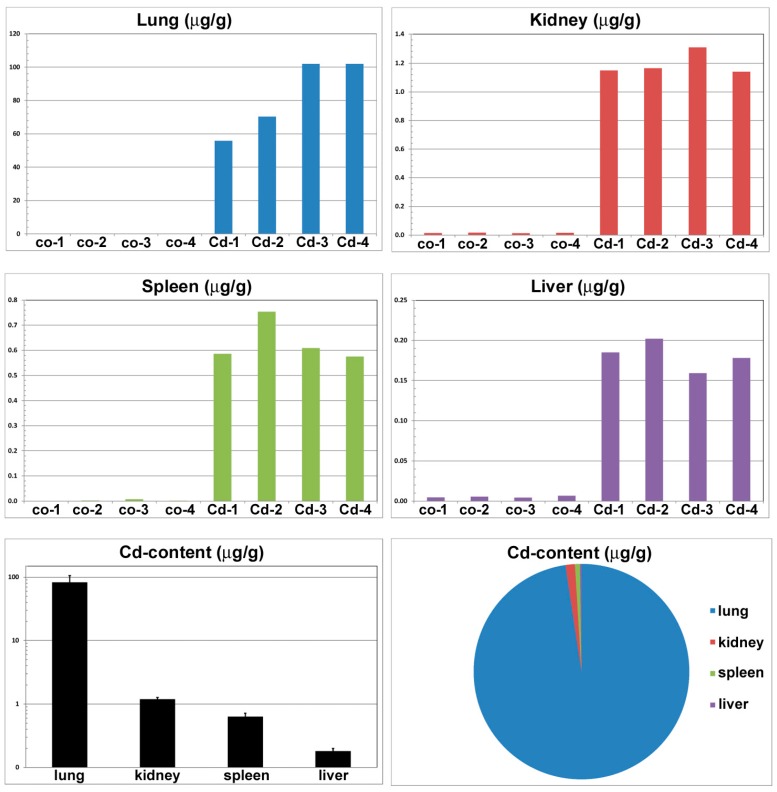
Content of cadmium in organs following six weeks exposure. Abbreviations in the *X* axis represent identification number of control mouse (co) or CdO nanoparticles inhalating mouse (Cd).

The content of cadmium in the organ of entry (lung) as well as in the secondary organs was markedly increased already after six weeks of nanoparticle inhalation (Figure 7). The content of cadmium in organs of exposed mice was more than 7500 times higher in lung, 140 times in kidney, 79 times in spleen and about 19 times higher in liver in comparison to the control animals. The highest accumulation of cadmium was found in lung, lower amount in kidney, spleen and liver, successively (Figure 7).

**Figure 8:**
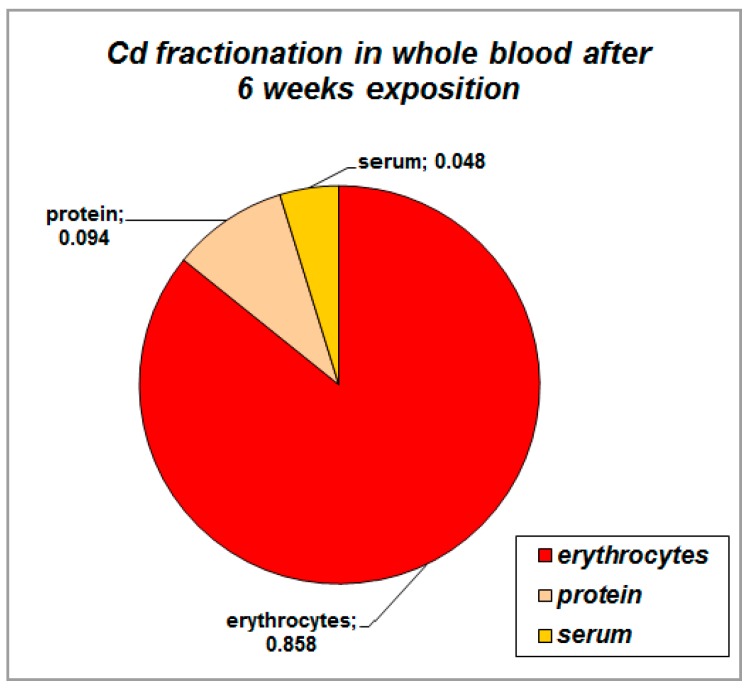
Cadmium content in individual blood fraction following six weeks exposure.

We also analyzed cadmium level in different fractions of blood (Figure 8). The largest proportion of cadmium in blood was found in blood element fraction (about 85.8% of cadmium), lower proportion in plasma protein fraction (9.4% of cadmium) and only small proportion was found in serum (about 4.8% of cadmium). The concentration of Cd in whole blood after six weeks exposure was 80 ng·g^−1^, whereas in reference mice blood was below limit of detection of 2 ng·g^−1^.

## 4. Discussion

In this study, we showed that inhalation of CdO nanoparticles results in their deposition not only in the organ of entry, the lungs, but also in the kidney and spleen, which are well known targets of cadmium toxicity. Major attention was given to subcellular distribution of CdO nanoparticles as well as to the pathologies caused at the cellular and subcellular level to the relevant tissue components.

Cadmium represents a common and important environmental contaminant, which was proven to be toxic and carcinogenic [20,21,22]. Food, industrial exposure and cigarette smoke are the major sources of cadmium. Tobacco smoke is significant source of Cd where 1 g of tobacco contains about 1–2 μg of cadmium or even more [23]. Dietary intake of cadmium varies from 10 to 40 μg per day to several hundred in polluted areas [24]. Concentration of cadmium in ambient air are generally low [8]. In countryside areas, an annual mean air cadmium concentration is only about 0.9 ng/m^3^ and, in the city, weekly mean concentrations of cadmium reach about 5 ng/m^3^ in Sweden [9]. In some urban areas, concentration of cadmium in respirable size Cd particles can reach up to 60 ng/m^3^ [25]. The European Union occupational exposure limits were set up on cadmium concentration in air on the level 5 ng/m^3^ [26]. In industrial areas, it was recommended to keep Cd concentration in the environment lower than 50 μg CdO/m^3^ in order to prevent cadmium-induced health diseases [27]. Therefore, we used in our experiments an average mass concentration of CdO nanoparticles about 32 µg CdO/m^3^ (estimated absorbed dose was 52 µg of CdO per gram of mouse body weight), which corresponds to Cd amount in some contaminated or industrial areas. We found that already this level of cadmium nanoparticles is causing serious microscopic and ultramicroscopic changes, especially in lung tissues.

### 4.1. Multiple Ways of Nanoparticle Uptake by Cells

Nanosized particles may enter the cells by various means, actively by phagocytosis, macropinocytosis, clathrin-mediated endocytosis, clathrin- and caveolae-independent endocytosis, caveolae-mediated endocytosis and by a passive movement through the plasma membrane with subsequent access to all subcellular compartments, including nucleus and mitochondria [28]. In our study, the uptake of CdO nanoparticles by the primary target cells was active, via endocytosis but not the macropinocytosis or clathrin-mediated endocytosis. All nanoparticles were found inside vesicular structures of variable size. For the secondary target organs, the way of entry remains rather obscures. Based on the presence of NPs in cytoplasmic vesicles, we may speculate about their transport via caveolae. However, some nanoparticles were freely distributed in the cytoplasm suggesting also passive entry across the plasma membrane.

### 4.2. Morphological Features of Nanoparticles in Cells—Are They Same or Different from Native Particles?

Only few studies described the changes occurring to nanoparticles during their passage through and among the cells. *In vitro* study [29] incubating metal nanoparticles Fe_2_O_3_, Y_2_O_3_ and ZnO with HAECs (human aortic endothelial cells) for 1–8 h revealed that Fe_2_O_3_ nanoparticles incorporated into HAECs maintained their faceted feature, ZnO particles exhibited same morphology as native ones, but Y_2_O_3_ particles lost the characteristic morphology of the original nanoparticles [29]. In addition, silica nanoparticles administered orally to rats changed their morphology and became irregular in kidney in contrast to liver where they stay intact and spherical as naive particles before their application [30]. The silica nanoparticles were sequestered into liver in their intact particulate forms but they slowly decomposed in kidneys [30]. In our study, CdO nanoparticles in cellular vesicles of primary target organ and in vesicles or membranous organelles of secondary target organs maintained their appearance.

### 4.3. Where Are Nanoparticles Located in Cells

Intracellular fate of nanoparticle has been previously studied using various models and particle chemistries. CeO_2_, Al_2_O_3_, FeO_x_ and TiO_2_ nanoparticles behaved about the same in *in vitro* cultured cells [31]. After 4 h of incubation, most particles were found on the extracellular surface of the cells. After 12 h, the nanoparticles appeared clearly inside endosomes and these structures moved together with its nanoparticle content towards the nuclear membrane but did not penetrate into the nucleus. Similarly to another study where human aortic endothelial cells incorporated Fe_2_O_3_, Y_2_O_3_ and ZnO nanoparticles [29], we also observed CdO particles integrated into cytoplasmic vesicles.

Katsnelson *et al.* assayed pulmonary phagocytosis of nanogold and nanosilver, with mean particle diameter of 50 and 49 nm, respectively, upon their intratracheal instillation [32]. Nanogold was found within phagosomes in the cytoplasm, typically as single particles and they were not in contact with any organelles. Gold nanoparticles were uniformly distributed throughout the cytoplasm and nucleus of alveolar macrophages. Particles were also seen inside the mitochondria in alveolar macrophages and neutrophilic leukocytes. Gold nanoparticles were seen in the nuclei of all cells examined. In contrast, silver nanoparticles were not found in every type of cells. Silver nanoparticles formed aggregates and they were frequently localized inside mitochondria either on their cristae or on the inner surfaces of its membranes. No silver nanoparticles have been revealed inside the nucleus in any of examined cells. Obviously, both the intracellular distribution of nanoparticles within phagocytosing cells and ultrastructural changes are not universal but are dependent on both their chemical nature and size.

### 4.4. Pathological Processes Initiated by Nanoparticles in Lungs

Previous study revealed that inhaled CdO nanoparticles caused acute inflammation and cell injury in lungs [33,34]. As acute effects of CdO particles have been previously examined in detail [16,35], we focused here on more detailed analysis on cellular and subcellular level and fate of nanoparticles in lung tissues following sub-chronic exposition. We showed that after six weeks of treatment, lungs of animals exposed to the CdO nanoparticles exhibited light hyperemia, focal hemorrhage, and alveolar emphysema, small areas of atelectasis, focal acute catarrhal bronchiolitis and also alveolitis in some animals. Pulmonary inflammation and associated lymphocyte and plasma cell invasion was found also around the small airways. Decrease of inflammation was observed following one week post exposure; however, it was still located around small airways [33]. We also observed the enhancement of bronchus associated lymphoid tissue in exposed mice and the number of foamy lipid laden macrophages increased in alveolar spaces. Similar processes of inflammation and cell injury were previously observed already shortly after inhalation of CdO nanoparticles [33,34]. Furthermore, thickening of alveolar septum with the presence of inflammatory cell infiltration inside the septum was similar to previous observations on rat lung tissue [33].

### 4.5. Pathological Processes Initiated by Nanoparticles in Liver

An increasing number of studies have shown that some nanomaterials are capable of distributing from the site of exposure (e.g., lungs and gut) to a number of secondary organs, including the liver [14,36]. The liver has been shown to be a preferential site of accumulation (>90% of nanoparticles compared with other organs), and alongside the kidneys, it may also be responsible for the clearance of nanoparticles from the blood [37].

Our findings of histopathological changes in liver are in consonance with previous study [14] describing liver tissue damage after intratracheal instillation of CeO_2_ nanoparticles in male rats. Similar to us they observed hydropic degeneration of the hepatocytes around the central vein region with sparing of the immediate periportal region, dilation of the sinusoids and portal inflammation, these changes were panlobular in nature. It is thought that hydropic degeneration can be caused by hypoxia, ischemia, or the treatment of hepatocytes with endotoxins or chemicals [14]. Similar pathological response of hepatocytes has also been observed following nasally instilled copper nanoparticles in mice [38].

Growing evidence indicates that mitochondrial dysfunction plays crucial roles in hepatotoxicity caused by cadmium [39,40]. Mitochondrial dynamics is regulated by the delicate balance of mitochondrial fusion and fission [41]. Exposure to cadmium induced the changes to mitochondrial morphology from elongated-tubular and filamentous networks to small and spherical structures both in L02 cells (human normal liver cell line) and in rat liver tissue. Mitochondrial fragmentation occurs as early as 3 h after exposure of L02 cells to CdCl_2_. Mitochondrial fragmentation not only precedes mitochondrial dysfunction but it is also required for mitochondrial dysfunction in the hepatotoxicity of cadmium [41]. Here, we observed the same amount of small spherical mitochondria in both cadmium-treated animals and in controls. Therefore, our observation cannot support the proposed link between hepatotoxicity of cadmium and the damage to mitochondria.

Some of these previous studies were focused only on hepatocytes but this cell type represent only about 80% of all liver cells. The liver contains numerous innate and adaptive immune cells—the single largest population of macrophages, the greatest densities of natural killer cells and natural killer T cells, and the largest reticuloendothelial cell network in the body [42]. Liver sinusoidal endothelial cells are critical for pathogen detection, capture and perhaps even antigen presentation; comprise about 50% of the non-parenchymal cells in the liver [42]. In some studies [43,44,45,46], the vascular endothelium was denoted as a target of cadmium toxicity. According to the vascular endothelial hypothesis, cadmium-induced degeneration of the hepatic endothelium produces a local ischemia that causes damage to surrounding hepatocytes. In our study, we clearly observed the signs of degeneration of liver endothelial cells. Liver sinusoidal endothelial cells were edematic and enlarged, while surrounding hepatocytes still showed normal architecture in most regions.

Kupffer cells represent about 35% of the non-parenchymal liver cells in normal adult mice [47]. Although primary injury to the liver results initially from direct effects of cadmium, secondary damage occurs from inflammatory processes that are initiated by the activation of Kupffer cells, which release a number of inflammatory mediators [45]. The early response proinflammatory cytokines activate other cells in the liver (endothelial cells, stellate cells, and hepatocytes) and induce the expression of chemokines that attract and activate the circulating inflammatory cells (neutrophils and monocytes) that lead to inflammation and further damage to the liver. We confirmed the presence of areas with periportal inflammation, with their number and size being increased with longer duration of cadmium exposure. Occasional neutrophilic granulocyte infiltrations around vessels were observed in some samples. Here, long term inhalation of CdO caused also occurrence of large areas of hepatic necrosis. Similar microscopic changes including hepatocyte degeneration together with large areas of hepatocytic necrosis were previously observed following sub-chronic (90 days) combined oral to lead and cadmium [36]. Our study did not demonstrate any changes in number of Kupffer cells, neither the cadmium nanoparticles were found in Kupffer cells.

Unexpectedly, we observed altered organelle content in Kupffer cells following nanoparticle exposure. Kupffer cells contained abundant smooth endoplasmic reticulum and free cytoplasmic ribosomes. Amount of their rough endoplasmic reticulum cisternae was decreased and mitochondria showed unusual arrangement of cristae. These findings are indicative of functional alteration from originally predominantly phagocytic cells to cells producing specific mediators that could maintain or enhance the inflammatory response to cadmium exposure.

### 4.6. Pathological Processes Initiated by Nanoparticles in the Kidney

Series of studies have previously demonstrated the kidney as a primary organ for cadmium accumulation following acute or chronic administration [48,49,50,51]. Cadmium was found to be efficiently retained in the kidney (half-time 10–30 years) and the concentration was proportional to that in urine [8]. Cadmium is nephrotoxic, initially causing kidney tubular damage. The tubular injury may progress to glomerular damage with decreased glomerular filtration rate, and eventually to renal failure [8].

Here, rather surprisingly, we did not find any significant changes in kidney. We only observed diffusely thickened filtration membrane with intramembranous electron dense deposits. Similarly to our study, rats exposed to Ag and Au nanometals demonstrated only moderate thickening and a clearly enhanced contour of the glomerular basal membranes [32].

Cadmium absorbed into the circulation is known to be rapidly taken up by the liver, where it is bound to metallothionein (MT), which is then slowly released back into circulation. The Cd-MT complex is freely filtered at the glomerulus and reabsorbed by the proximal tubule. MT-bound Cd has a very long half-life, anywhere between 10 and 30 years for humans, so it accumulates slowly over many years [52]. Therefore, it is possible that six weeks of exposure used here was not long enough to reach harmful level of cadmium in the kidney.

### 4.7. Blood as a Transporter of Cadmium Nanoparticles to Target Organs

Only handful of studies addressed the issue of cadmium transport by blood. It was proposed that Cd ions, rather than intact nanoparticles, are transported from the site of deposition into target organs. It was shown that water-insoluble CdO may still be dissolved in the microenvironment of the lung so that the Cd ions are released and transported via the blood to other tissues [53].

Our study is the first one comparing the content of cadmium in individual fractions of whole blood. We have found that about 86% of total blood cadmium is contained in its element fraction. Similarly to us, approximately 50% of cadmium was found to be distributed among the cellular constituents of blood, mainly in the erythrocytes blood previously [52]. The uptake of cadmium by erythrocytes was proposed to be mediated by an anion exchanger in their plasma membrane [52]. We also found that about 9% of cadmium was in plasma fraction and only very small amount was in serum. Previously, albumin was found to be an important carrier of cadmium in the circulation [54] as well as other proteins, peptides or amino acids such as transferrin and low molecular weight thiols like cysteine and glutathione were mentioned as low affinity carriers [55].

Hematopoiesis is one of the most sensitive processes to assess the toxicity of drugs in humans and animals. It was indicated that the combination of lead and cadmium in low doses [56] can cause varying degrees of inflammatory injury in rats. Moreover, significantly decreased level of red blood cell count, hemoglobin, hematocrit, mean corpuscular volume, mean corpuscular hemoglobin, and mean corpuscular hemoglobin concentration indicated microcytic hypochromic anemia of rats. In addition, decreased number of platelets was observed after exposure. In our study, small increase of hematopoietic activity with elevated percentage of reticulocytes was seen in the spleen and also slightly increased amount of megakaryocytes was observed in red pulp. Thus, although we did not examine hematological parameters *per se*, our histological findings fit the overall scheme presented by others.

## 5. Conclusions

Nanoparticles of various chemistries and sizes are becoming a reality in many industrial applications. As a result, there is an increasing need for understanding the adverse effects that nanoparticles may have on human and animal health. To this point, here we have shown that cadmium nanoparticles indeed exhibit serious negative effects on cellular and subcellular levels similar to other toxic metals. Since cadmium nanoparticles are transported into the secondary organs via blood circulation, the exposure to high doses of such nanoparticles leads to complex symptoms affecting the entire body.

## Supplementary Materials

Supplementary materials can be found at http://www.mdpi.com/1422-0067/17/6/874/s1.
