# Supplementary material for: Inhaled Cadmium Oxide Nanoparticles: Their in Vivo Fate and Effect on Target Organs
Source: Int J Mol Sci. 2016 Jun 3;17(6):874. doi: 10.3390/ijms17060874 (PMC4926408; doi:10.3390/ijms17060874)
Supplement: Supplementary file 1 [file ijms-17-00874-s001.pdf]

Supplementary Materials: Inhaled Cadmium Oxide Nanoparticles: Their *in Vivo* Fate and Effect on Target Organs

Jana Dumkova, Lucie Vrlikova, Zbynek Vecera, Barbora Putnova, Bohumil Docekal, Pavel Mikuska, Petr Fictum, Ales Hampl and Marcela Buchtova

The estimation of deposited dose was calculated based on previously published methodology [17,18] and base on the average mass concentration of CdO nanoparticles (31.7 μg CdO/m^3).

$$\text{Deposited dose} = (C \times \text{RMV} \times T \times \text{DF})/\text{BW}$$

where C is average concentration in the exposure atmosphere 31.7 μg CdO/m^3. RMV is respiratory minute volume (RMV = 0.499 × BW × 0.809 L/min [17]). T is exposure time (min) = 60,480 (6 × 7 × 24 × 60). DF is pulmonary deposition fraction (10%), therefore 0.1 [19]. BW is average body weight (g) = 24 g. Final deposited = 52 μg/g.

Estimated dose of CdO was 52 μg per gram of mouse body weight over the 6 weeks inhalation period.

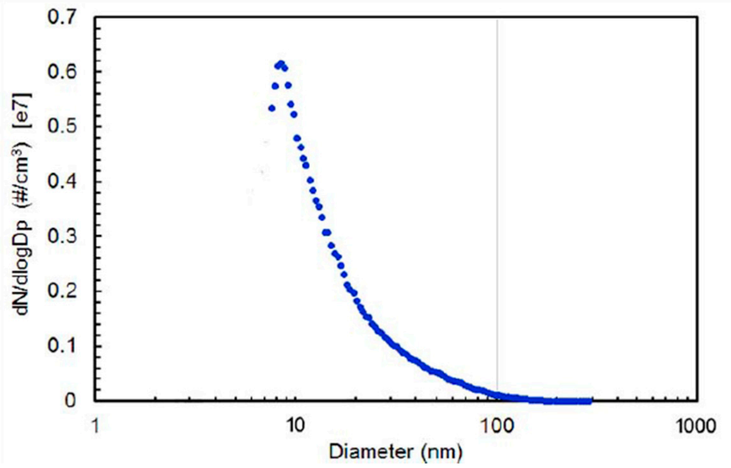

Figure S1. The size distribution of CdO nanoparticles in the size range 7.64–229.6 nm (measured in inhalled air with the SMPS, model 3936L72).

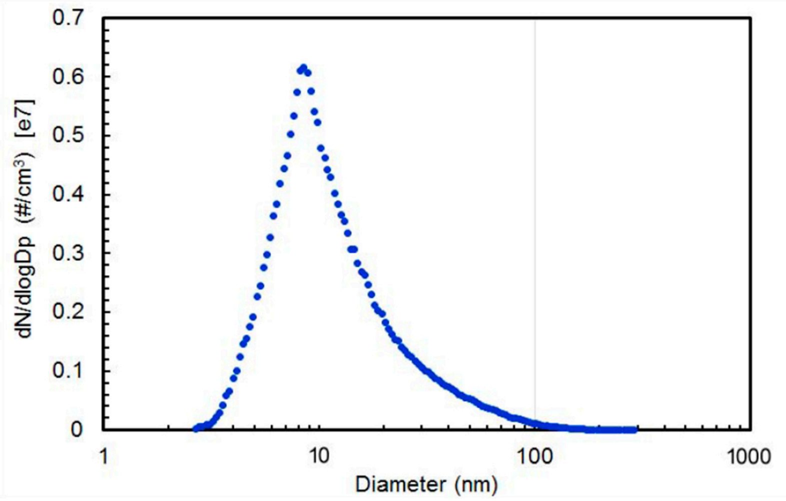

Figure S2. The combined size distribution of CdO nanoparticles in the size range 2.02–229.6 nm (measured in inhalled air with the SMPS, DMA model 3081 + nanoDMA model 3085).

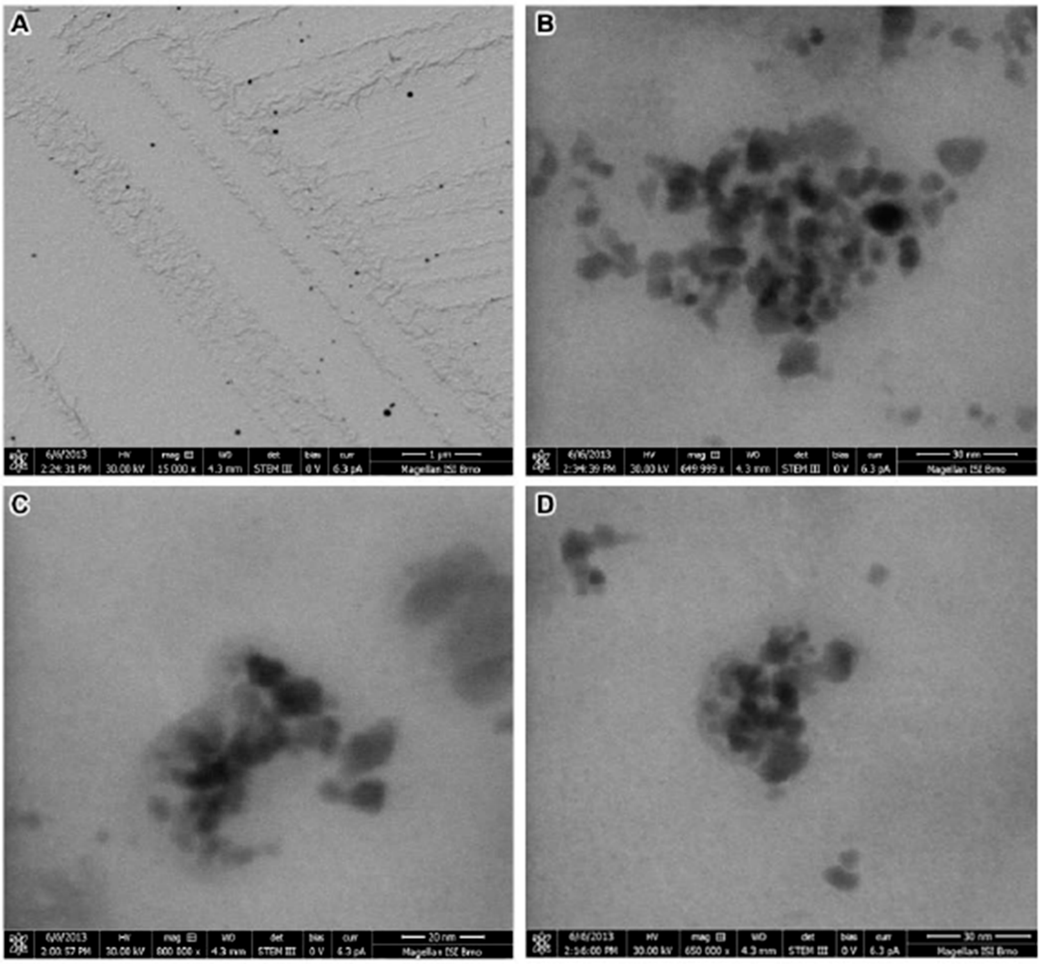

Figure S3. CdO nanoparticles in electron microscope (**A**–**D**).
